# Effectiveness and Adverse Effects of Risperidone in Children with Autism Spectrum Disorder in a Naturalistic Clinical Setting at a University Hospital in Oman

**DOI:** 10.1155/2022/2313851

**Published:** 2022-01-31

**Authors:** Salim Al-Huseini, Ali Al-Barhoumi, Miad Al-Balushi, Amira Al-Hosni, Tamadhir Al-Mahrouqi, Balqees Al-Mahrizi, Sanjay Jaju, Hassan Mirza

**Affiliations:** ^1^Psychiatry Residency Training Program, Oman Medical Specialty Board, Muscat, Oman; ^2^Colleges of Medicine & Health Sciences, Sultan Qaboos University, Muscat, Oman; ^3^Departments of Behavioral Medicine, Sultan Qaboos University Hospital, Muscat, Oman; ^4^Department of Family Medicine and Public Health, College of Medicine & Health Sciences, Sultan Qaboos University, Muscat, Oman

## Abstract

**Objective:**

This study aimed at examining the effectiveness of treating children with autism spectrum disorder (ASD) who present with irritability, aggression, and disruptive behavior at the Sultan Qaboos University Hospital (SQUH) in Muscat, Oman, with risperidone, and to note any sex-based differences among this cohort.

**Method:**

This was a retrospective study conducted at the Department of Behavioral Medicine at SQUH over two years from January 2017 to December 2018. This study included all children aged 3 to 18 years attending the Child and Adolescent Mental Health Service (CAMHS) outpatient clinic with a diagnosis of ASD, based on the DSM-5 criteria, and comorbid disruptive behavior, who had been prescribed risperidone.

**Result:**

This study identified 95 ASD patients (72 males). Male patients' BMI score after 12 months of risperidone treatment showed an increase by 0.62 (1.57 SD; *P*=0.001); however, there was no significant change among female patients. Somnolence was noted in 69.6% of female patients as compared to 34.7% of males (*P*=0.003). Among those with a family history of ASD, 5 out of 17 patients had treatment success (29.4%), whereas 70 out of 78 patients (90.0%) who did not have a similar history had successful treatment.

**Conclusion:**

In conclusion, low-dose risperidone monotherapy is effective and well tolerated among some children with ASD who present with disruptive behavior in a naturalistic clinical setting. However, we found that some of the side effects, such as weight gain and somnolence, were concerning.

## 1. Introduction

Autism spectrum disorders (ASD) are a group of complex neurodevelopment disorders that are characterized by abnormal or delayed speech, poor social communication, repetitive stereotypical behavior/restricted interests, and sensory abnormalities [1]. The prevalence of ASD has risen globally over the past few decades, which has mostly been attributed to changes in reporting practices [2]. The current prevalence of ASD in the developed world has been estimated to be at least 1.5% [3–5].

In Oman, the current research suggests that the prevalence of ASD is 20.35 cases per 10,000 children [6]. The etiology of ASD is multifactorial, with genetic, environmental, and prenatal factors all being regarded as major contributing risk factors [7–9]. Psychiatric comorbidities, such as depression, anxiety, obsessive-compulsive disorder, and attention deficit hyperacidity disorder, are common in patients diagnosed with ASD, and it has been estimated that 70.0% suffer from at least one comorbid disorder, with 40.0% having two or more [10]. Patients with ASD can also present with associated behavioral symptoms, such as aggression and irritability, which can result in significant harm to those affected, as well as cause marked distress for their families and primary caregivers [11, 12].

Although there is no efficient pharmacological treatment for the core symptoms of ASD [13], atypical antipsychotics, such as risperidone and aripiprazole, are U.S. Food and Drug Administration (FDA)-approved for the management of ASD-associated irritability [14, 15]. The use of antipsychotics in the management of ASD-associated irritability and aggression has been frequently prescribed as an add-on to behavioral management strategies [16]. A systemic review and meta-analysis on the use of atypical antipsychotics in managing irritability in children with ASD concluded that both risperidone and aripiprazole significantly reduced irritability, and both drugs had comparable efficacy and safety profiles in children with ASD [17]. However, although atypical antipsychotics are the preferred choice compared to typical antipsychotics used by the older generation, their long-term use, just like in the adult population, has been associated with side effects, such as sedation and weight gain [18–20].

Most of the available literature addressing the use of atypical antipsychotic medications in managing irritability and aggression associated with ASD comes from non-Middle Eastern populations, and, to the best of our knowledge, no studies from Oman have looked into the effectiveness and side effect profiles of pharmacological interventions in this group of patients. In Oman, mental health services in general, and child and adolescent mental health services in particular, are scarce [21]. Medication use is one of the main management tools due to the lack of specialized canters for children with ASD and lack of healthcare and educational programs or related services [22].

In this study, among children with ASD who have undergone a minimum of one year of treatment with risperidone, we aim at examining the association of effectiveness of treatment, as well as the association of other factors (including age, indications for treatment with risperidone, family history, and side effect profile of risperidone), by sex in children with ASD who present with irritability, aggression, and disruptive behavior at Sultan Qaboos University Hospital (SQUH) in Oman. We also propose to study the different factors associated with treatment effectiveness.

## 2. Methods

This was a retrospective cohort study to evaluate the effectiveness and adverse effects associated with the use of risperidone on children with a diagnosis of ASD who presented with irritability at the Department of Behavioral Medicine in SQUH over two years, from January 2017 to December 2018.

This study included all children aged 3 to 18 years, attending the Child and Adolescent Mental Health Service (CAMHS) outpatient clinic with a diagnosis of ASD based on the DSM-5 criteria, who had comorbid disruptive behavior and had been prescribed risperidone. Each participant had at least one year of follow-up after being prescribed risperidone. Children with comorbid epilepsy and those on anticonvulsants, as well those prescribed any other psychotropic medications, were excluded. The medical record number of each patient with a diagnosis of ASD was accessed through the hospital health information system, and children who had been diagnosed with comorbid disruptive behavior and prescribed risperidone were included in the study. The following demographic variables were included: age, sex, family history, average dosage of risperidone, and duration of treatment.

For children and adolescents, body mass index (BMI) is not a diagnostic tool. Instead, it is used to screen for potential weight and health-related issues. The BMI is interpreted differently for children and adolescents even though it is calculated the same way as it is for adults. Due to changes in weight and height with age, as well as its relationship to body fat, BMI levels among children and adolescents are expressed relative to other children of the same sex and age [23]. Since we aimed at determining the change in the BMI in each subject after being on risperidone treatment for a minimum of 12 months, the BMI at the start of treatment and after 12 months of treatment was documented.

The effectiveness of the pharmacological treatment was assessed based on the clinician's notes and the clinical global score (CGI), as well as feedback given by the patients or their primary caregivers during follow-up visits. The CGI scale is a well-established research rating tool, applicable to all psychiatric disorders, which is routinely used by practicing clinicians to assess a patient's global functioning before and after initiating a medication. The CGI has two components: the CGI-Severity (CGI-S), which rates illness severity, and the CGI-Improvement (CGI-I), which rates change from the initiation (baseline) of treatment [24].

CGI-S rates the patient's illness severity on a seven-point scale: 1 = normal, not at all ill; 2 = borderline mentally ill; 3 = mildly ill; 4 = moderately ill; 5 = markedly ill; 6 = severely ill; and 7 = among the most extremely ill patients. This rating is based upon observed and reported symptoms, behavior, and function noted in the patients in seven days prior. The CGI-I compares the patient's overall clinical condition to the baseline assessment and rates improvement on a similar seven-point scale: 1 = very much improved since the initiation of treatment; 2 = much improved; 3 = minimally improved; 4 = no change from baseline (the initiation of treatment); 5 = minimally worse; 6 = much worse; and 7 = very much worse since the initiation of treatment. Any side effects experienced by the children from the medications were retrieved from their clinical notes, as well as any routine lab investigations, where applicable.

### 2.1. Statistical Analysis

Due to the biological differences between males and females who were in the developmental phase of life in this study, the sex-based outcomes and also outcomes within male and female groups were analyzed. The collected data were revised, coded, tabulated, and analyzed using the Statistical Package for the Social Sciences (SPSS), version 23 (IBM Corp, Armonk, NY, USA). The sociodemographic variables were categorized and their frequency and proportions were stated. The continuous variables were reported as mean and standard deviation (SD) and tested by independent samples *t*-test or paired *t*-test as appropriate. All other demographic variables and those related to effectiveness and adverse effects were reported as proportions. The chi-square test was used to test the significance of association between gender and age groups, indications for treatment, family history, treatment improvement according to CGI scale, number of side effects, and individual side effects. The chi-square test was also used to determine the association of effectiveness of treatment in age groups and by sex, family history, and side effects of risperidone. A *P* value of <0.05 was considered statistically significant.

### 2.2. Ethical Approval

Ethical approval was granted by the College of Medicine and Health Sciences at SQUH, Muscat, Oman (MREC #1707). The study adhered to the Declaration of Helsinki and the American Psychological Association guidelines with regard to ethical human research, including confidentiality, privacy, and data management.

## 3. Results

This study identified 95 patients with ASD (72 males and 23 females) from those who presented during the study period and who had at least one year of follow-up after being initiated on risperidone treatment.  Table 1 shows the mean ± SD age at starting risperidone was 9.47 ± 3.66 years. The mean ± SD dose of risperidone was 1.04 ± 0.91 mg/day with the range of 0.25 to 6 mg/day. The mean ± SD duration of the treatment with risperidone was 38.6 ± 28.8 months.

There was no significant difference between the male and female patients' ages (*P*=0.115), their dose of risperidone (*P*=0.954), and their duration of treatment (*P*=0.073).

There was an increased mean change in the BMI score after 12 months of treatment with risperidone in the male group (0.62 [±1.57]; *P*=0.001), with no significant change among females (Table 2).

In Table 3, the sex-based association some side effects of risperidone are noted. Somnolence was noted in 69.56% of female patients, as compared to 34.72% of male patients (*P*=0.003). There were no significant sex-based differences across the age groups, indications for treatment with risperidone, family history, the effectiveness of treatment, and side effect profiles of risperidone, namely the total number of side effects and other side effects, such as extrapyramidal symptoms, sedation, and hyperprolactinemia.

The criteria of treatment success and failure were based on the clinician's notes and the clinical global score (CGI). Effectiveness was evaluated using the Clinical Global Impression Scale Improvement (CGI-I) score and the mean change from baseline in the Clinical Global Impression Scale Severity of Illness (CGI-S) score. In the present study, the CGI-I scores of 1 (very much improved) and 2 (much improved) were grouped as treatment success, whereas the rest of the CGI-I scores was grouped as treatment failure. Mean CGI-I scores showed improvement during the course of the treatment; at baseline, the mean CGI-I score was 2.11 and at 12 months of treatment the mean CGI-I score was 3.85. Mean changes from baseline in CGI-I and is shown in Table 4. Significant improvement was observed in the score of CGI-I (3.85 ± 0.36, *P* < 0.001).

The relevant factors associated with the effectiveness of treatment are highlighted in Table 5.

Among those who had a family history of ASD, 5 out of 17 patients showed improvement with treatment (29.4%), whereas 70 out of 78 patients (90%) who did not have a similar history showed had effective treatment. Among those who showed the effectiveness of treatment, it was noted that 5 out of 75 patients (6.6%) had a positive family history of ASD, but among those who showed treatment failure, 12 out of 20 (60%) had a positive family history (*P* < 0.001). Other factors based on the age groups, sex-based differences, and specific side effects were not associated with the effectiveness of treatment.

Associations of each of the adverse effects with the dose of risperidone were assessed (Table 6). A mean difference in the dose of risperidone in association with some side effects of risperidone was noted. Hyperprolactinemia was significantly associated with an increased dose of risperidone (*P*=0.048). However, there was no significant correlation noted between the dose of risperidone and other adverse effects like extrapyramidal symptoms, sedation, and weight gain.

## 4. Discussion

This is the first study from Oman on children clinically diagnosed with ASD with comorbid challenging behavior requiring treatment with risperidone. In this study, the majority of patients were male, with a male: female ratio of approximately 3 : 1 [25], which is lower than the globally assumed ratio of male: female ASD diagnosis ratio of 4 : 1 [26]. Conversely, a recent systemic review and meta-analysis concluded that the true male: female ratio is closer to 3 : 1, consistent with the findings from this study.

The mean dose of risperidone prescribed to the patients in this study was similar among both the male and female patients (1 mg/day) with no statistical difference, which is close to the dose of risperidone proven to be effective among children with ASD and disruptive behavior [27–30]. Several studies have shown the effectiveness of risperidone in reducing the disruptive behavior associated with ASD, with an overall positive response rate of 70% [31]. Similarly, the patients in this study showed a reduction in aggression, irritability, and hyperactivity. According to the CGI-I scale, a total of 59 male patients (82%) and 16 female patients (70%) improved, resulting in a positive overall response rate in 75 patients (79%).

With regard to the clinical and demographic factors associated with the effectiveness of risperidone, age, sex, and side effects were not statistically significant. However, patients with no family history of ASD were more likely to respond to risperidone treatment. This association could be explained by the notion that patients with a family history of autism having a more severe and complex phenotypic presentation of the disorder [32, 33]. In our cohort of children with ASD, 17% had a family history of ASD. Genetic disorders are of particular concern in Oman, as more than 50% of marriages in the country are consanguineous, with 39% being between first cousins, putting Oman at a significantly higher risk of inherited disorders [34–36]. The finding that ASD patients without a family history had a better response to treatment with risperidone than those with a family and genetic history is consistent with the theory that more severe cases of ASD pose a challenge concerning treatment response. This severe end of the autism spectrum, for whom assessment and treatment pose a particular challenge, is arguably the least well-understood, making such cases underrepresented in treatment studies [37]. Other factors, such as sex, age, and presence of adverse events, were not significantly associated with an effective response to treatment.

With regard to the side effects associated with risperidone use, weight gain and somnolence were the most reported. There are a plethora of studies that have concluded that weight gain and subsequent metabolic consequences are a side effect of risperidone monotherapy in children with ASD [18, 38, 39]. The results from the current study showed that there was an increase in the BMI after 12 months of treatment with risperidone. However, this rise in the BMI was only statistically significant for male patients. Similarly, somnolence was reported in 43% of the children who received risperidone monotherapy, which was statistically significant in one-third of males and two-third of females. This is in line with the findings of various studies that have reported somnolence as one of the most common side effects associated with risperidone [18, 38, 40]. Other side effects linked to risperidone, such as extrapyramidal symptoms and hyperprolactinemia, were reported in 8.4% and 9.4% of patients, respectively, and were not statistically significant between genders. However, when we assessed the associations of each of the adverse effects with the dose of risperidone, we found that only hyperprolactinemia has significant correlations with risperidone dose. This could be due to the low dose of risperidone prescribed in this study, and these adverse effects are usually dose-dependent [41, 42].

The strength of this study was that it examined all children with ASD with comorbid disruptive behavior attending the CAMHS at SQUH, and it is the first study from an Arabian Gulf region to scrutinize the effectiveness and side effects of risperidone in such a cohort of patients. The limitations are the relatively small sample size and the high chance that children with ASD might have been missed if they did not have access to the tertiary hospital service.

## 5. Conclusion

We have demonstrated that low-dose risperidone monotherapy is effective and well tolerated in some children with ASD, presenting with disruptive behavior, in a naturalistic clinical setting. However, weight gain and somnolence were concerning side effects. However, this rise in the BMI was only statistically significant for male patients. Therefore, the decision to prescribe risperidone must be thoroughly discussed with caregivers and should be initiated after weighing the benefits and risks on a case-by-case basis, and after all alternative nonpharmacological interventions have been exhausted. Due to the small sample size, therefore, a future follow-up study on this subject is needed with a larger sample size.

## Figures and Tables

**Table 1:** Baseline descriptive statistics by sex of children with autism spectrum disorder who had at least one year of follow-up after being initiated on risperidone treatment.

Variable	Males (*n* = 72), *Mean (SD*)	Females (*n* = 23), *Mean (SD)*	*T*-test *P* value
Mean (SD) age at starting risperidone, years = **9.47 (3.66)**	9.14 (3.67)	10.52 (3.50)	0.115
Mean (SD) dose of risperidone, mg/day = **1.04 (0.91)**	1.05 (0.93)	1.03 (0.85)	0.954
Mean (SD) duration of treatment, months = **38.57 (28.81)**	36.31 (21.07)	45.65 (23.03)	0.073

SD = standard deviation.

**Table 2:** Sex-specific changes in the body mass index of children with an autism spectrum disorder in the 12 months' postinitiation of treatment with risperidone, as compared to baseline.

Sex	BMI factor	Mean (SD)	Paired *t*-test *P* value
Male	BMI at the start of treatment	15.80 (2.42)	—
	BMI 12 months after treatment	16.43 (3.15)	
	Change in BMI after 12 months of treatment	0.62 (1.57)	**0.001**

Female	BMI at the start of treatment	16.77 (3.63)	—
	BMI 12 months after treatment	16.97 (3.86)	
	Change in BMI after 12 months of treatment	0.20 (2.83)	0.738

BMI body mass index. Bold indicates that *P* value of <0.05 is considered statistically significant.

**Table 3:** Association of baseline factors, the effectiveness of treatment, and side effects with the sex of children with autism spectrum disorder receiving risperidone treatment for a minimum of 12 months.

Variable		Male	Female	Chi-square *P* value
Age groups (years)	<12	55	12	0.258
	≥12	17	11	
Indications for treatment with risperidone	Aggression	44	14	0.963
	Disruptive behavior	8	3	
	Hyperactivity	20	6	
Family history	Yes	12	5	0.581
	No	60	18	
Effectiveness (treatment success according to CGI scale)	Improved	60	17	0.205
	Failure	12	6	
Somnolence	Yes	25	16	**0.003**
	No	47	7	
Extra pyramidal symptoms	Yes	6	2	0.957
	No	66	21	
Hyperprolactinemia	Yes	6	3	0.502
	No	66	20	

CGI clinical global score. Bold indicates that *P* value of <0.05 is considered statistically significant.

**Table 4:** Mean CGI-I score of children with an autism spectrum disorder in the 12 months' postinitiation of treatment with risperidone, as compared to baseline.

CGI-I scores	Mean (SD)	Paired *t*-test *P* value
Mean CGI-I scores at the start of treatment	2.11 (0.75)	
Mean CGI-I scores at 12 after treatment	3.85 (0.36)	
Change in the mean CGI-I scores after 12 months of treatment	−1.75 (0.90)	**0.001**

**Table 5:** Association of the effectiveness of risperidone treatment in children with autism spectrum disorder receiving treatment for minimum of 12 months with baseline factors and side effect profile.

Variable		Effectiveness of treatment		Chi-square *P* value
		Improved	Failure	
Age (years)	<12	52	15	0.621
	≥12	23	5	
Sex	Male	60	12	**0.205**
	Female	17	6	
Family history	Yes	5	12	**<0.001**
	No	70	8	
Impulsivity	Yes	31	6	**0.356**
	No	44	14	
Somnolence	Yes	31	10	**0.487**
	No	44	10	
Extra pyramidal symptoms	Yes	8	0	**0.127**
	No	67	20	
Hyperprolactinemia	Yes	7	2	0.928
	No	68	18	

Bold indicates that *P* value of <0.05 is considered statistically significant.

**Table 6:** Association of mean dose of risperidone treatment in children with autism spectrum disorder with adverse effects.

Variable side affect		Dose of risperidone, mg/day mean (SD)	*P* value
Somnolence	Yes	1.07 (0.82)	0.68
	No	1.01 (0.97)	
Extra pyramidal symptoms	Yes	1.16 (0.81)	0.95
	No	1.03 (0.91)	
Hyperprolactinemia	Yes	1.36 (1.29)	0.048
	No	1.01 (0.86)	
Weight gain	Yes	1.05 (0.85)	0.988
	No	1.03 (0.97)	

## Data Availability

Data are available from the corresponding author on reasonable request.

## References

[1] Association A. P. (2013). *Diagnostic and Statistical Manual of Mental Disorders*.

[2] Hansen S. N., Schendel D. E., Parner E. T. (2015). Explaining the increase in the prevalence of autism spectrum disorders. *JAMA Pediatrics*.

[3] Baxter A. J., Brugha T. S., Erskine H. E., Scheurer R. W., Vos T., Scott J. G. (2015). The epidemiology and global burden of autism spectrum disorders. *Psychological Medicine*.

[4] Christensen D. L., Braun K. V. N., Baio J. (2018). Prevalence and characteristics of autism spectrum disorder among children aged 8 Years - autism and developmental disabilities monitoring network, 11 sites, United States, 2012. *MMWR. Surveillance Summaries*.

[5] Lyall K., Croen L., Daniels J. (2017). The changing epidemiology of autism spectrum disorders. *Annual Review of Public Health*.

[6] Al-Mamari W., Idris A. B., Dakak S. (2019). Revisiting the prevalence of autism spectrum disorder among Omani children: a multicentre study. *Sultan Qaboos University Medical Journal [SQUMJ]*.

[7] Hallmayer J., Cleveland S., Torres A., Phillips J., Cohen B., Torigoe T. (2011). Genetic heritability and shared environmental factors among twin pairs with autism. *Archives of General Psychiatry*.

[8] Risch N., Hoffmann T. J., Anderson M., Croen L. A., Grether J. K., Windham G. C. (2014). Familial recurrence of autism spectrum disorder: evaluating genetic and environmental contributions. *American Journal of Psychiatry*.

[9] Bauman M. L., Kemper T. L. (2005). Neuroanatomic observations of the brain in autism: a review and future directions. *International Journal of Developmental Neuroscience*.

[10] Simonoff E., Pickles A., Charman T., Chandler S., Loucas T., Baird G. (2008). Psychiatric disorders in children with autism spectrum disorders: prevalence, comorbidity, and associated factors in a population-derived sample. *Journal of the American Academy of Child & Adolescent Psychiatry*.

[11] Volkmar F. R., Lord C., Bailey A., Schultz R. T., Klin A. (2004). Autism and pervasive developmental disorders. *Journal of Child Psychology and Psychiatry*.

[12] Chavez B., Chavez-Brown M., Rey J. A. (2006). Role of risperidone in children with autism spectrum disorder. *Annals of Pharmacotherapy*.

[13] Farmer C., Thurm A., Grant P. (2013). Pharmacotherapy for the core symptoms in autistic disorder: current status of the research. *Drugs*.

[14] DeFilippis M., Wagner K. D. (2016). Treatment of autism spectrum disorder in children and adolescents. *Psychopharmacology Bulletin*.

[15] Anagnostou E., Zwaigenbaum L., Szatmari P. (2014). Autism spectrum disorder: advances in evidence-based practice. *CMAJ Canadian Medical Association*.

[16] Posey D. J., Stigler K. A., Erickson C. A., McDougle C. J. (2008). Antipsychotics in the treatment of autism. *Journal of Clinical Investigation*.

[17] Fallah M. S., Shaikh M. R., Neupane B., Rusiecki D., Bennett T. A., Beyene J. (2019). Atypical antipsychotics for irritability in pediatric autism: a systematic review and network meta-analysis. *Journal of Child and Adolescent Psychopharmacology*.

[18] Boon-Yasidhi V., Jearnarongrit P., Tulayapichitchock P., Tarugsa J. (2014). Adverse effects of risperidone in children with autism spectrum disorders in a naturalistic clinical setting at siriraj hospital, Thailand. *Psychiatry Journal*.

[19] Rasimas J. J., Liebelt E. L. (2012). Adverse effects and toxicity of the atypical antipsychotics: what is important for the pediatric emergency medicine practitioner?. *Clinical Pediatric Emergency Medicine*.

[20] Kirino E. (2014). Efficacy and tolerability of pharmacotherapy options for the treatment of irritability in autistic children. *Clinical Medicine Insights: Pediatrics*.

[21] Mirza H. (2018). Child and adolescent mental health services in Oman. *London Journal of Primary Care*.

[22] Alakhzami M., Ann H. (2020). Individuals with autism spectrum disorders and developmental disorders in Oman: an overview of current status. *Journal of Autism and Developmental Disorders*.

[23] CDC (2021). *About Child & Teen BMI, Healthy Weight, Nutrition, and Physical Activity*.

[24] Busner J., Targum S. D. (2007). The clinical global impressions scale: applying a research tool in clinical practice. *Psychiatry (Edgmont (Pa.: Township))*.

[25] Fombonne E. (2003). Epidemiological surveys of autism and other pervasive developmental disorders: an update. *Journal of Autism and Developmental Disorders*.

[26] Loomes R., Hull L., Mandy W. P. L. (2017). What is the male-to-female ratio in autism spectrum disorder? A systematic review and meta-analysis. *Journal of the American Academy of Child & Adolescent Psychiatry*.

[27] Shea S., Turgay A., Carroll A. (2004). Risperidone in the treatment of disruptive behavioral symptoms in children with autistic and other pervasive developmental disorders. *Pediatrics*.

[28] Research Units on Pediatric Psychopharmacology Autism Network (2005). Risperidone treatment of autistic disorder: longer-term benefits and blinded discontinuation after 6 months. *The American Journal of Psychiatry*.

[29] McDougle C. J., Scahill L., Aman M. G. (2005). Risperidone for the core symptom domains of autism: results from the study by the autism network of the research units on pediatric psychopharmacology. *American Journal of Psychiatry*.

[30] Luby J., Mrakotsky C., Stalets M. M. (2006). Risperidone in preschool children with autistic spectrum disorders: an investigation of safety and efficacy. *Journal of Child and Adolescent Psychopharmacology*.

[31] Maneeton N., Maneeton B., Putthisri S., Woottiluk P., Narkpongphun A., Srisurapanont M. (2018). Risperidone for children and adolescents with autism spectrum disorder: a systematic review. *Neuropsychiatric Disease and Treatment*.

[32] Polyak A., Rosenfeld J. A., Girirajan S. (2015). An assessment of sex bias in neurodevelopmental disorders. *Genome Medicine*.

[33] Pizzo L., Jensen M., Polyak A. (2019). Rare variants in the genetic background modulate cognitive and developmental phenotypes in individuals carrying disease-associated variants. *Genetics in Medicine*.

[34] Islam M. M. (2012). The practice of consanguineous marriage in Oman: prevalence, trends and determinants. *Journal of Biosocial Science*.

[35] Hamamy H., Antonarakis S. E., Cavalli-Sforza L. L. (2011). Consanguineous marriages, pearls and perils: geneva international consanguinity workshop report. *Genetics in Medicine*.

[36] Al-Kindi R. M., Kannekanti S., Natarajan J., Shakman L., Al-Azri Z., Al-Kalbani N. I. (2019). Awareness and attitude towards the premarital screening programme among high school students in Muscat, Oman. *Sultan Qaboos University Medical Journal*.

[37] Stedman A., Taylor B., Erard M., Peura C., Siegel M. (2019). Are children severely affected by autism spectrum disorder underrepresented in treatment studies? An analysis of the literature. *Journal of Autism and Developmental Disorders*.

[38] Lemmon M. E., Gregas M., Jeste S. S. (2011). Risperidone use in autism spectrum disorders: a retrospective review of a clinic-referred patient population. *Journal of Child Neurology*.

[39] Scahill L., Jeon S., Boorin S. J. (2016). Weight gain and metabolic consequences of risperidone in young children with autism spectrum disorder. *Journal of the American Academy of Child & Adolescent Psychiatry*.

[40] Aman M. G., Arnold, M.D. L. E., McDougle C. J. (2005). Acute and long-term safety and tolerability of risperidone in children with autism. *Journal of Child and Adolescent Psychopharmacology*.

[41] Bishop J. R., Pavuluri M. N. (2008). Review of risperidone for the treatment of pediatric and adolescent bipolar disorder and schizophrenia. *Neuropsychiatric Disease and Treatment*.

[42] Peuskens J., Pani L., Detraux J., De Hert M. (2014). The effects of novel and newly approved antipsychotics on serum prolactin levels: a comprehensive review. *CNS Drugs*.

